# Cordycepin Accelerates Osteoblast Mineralization and Attenuates Osteoclast Differentiation In Vitro

**DOI:** 10.1155/2018/5892957

**Published:** 2018-10-16

**Authors:** Su-Bin Yu, Hye-Jin Kim, Hae-Mi Kang, Bong-Soo Park, Ji-Hye Lee, In-Ryoung Kim

**Affiliations:** ^1^Department of Oral Anatomy, School of Dentistry, Pusan National University, Busandaehak-ro 49, Mulguem-eup, Yangsan-si, Gyeongsangnam-do 50612, Republic of Korea; ^2^Department of Dental Hygiene, Dong-Eui University, 176 Eomgwang-ro, Busanjin-gu, Busan 47340, Republic of Korea; ^3^BK21 PLUS Project, School of Dentistry, Pusan National University, Busandaehak-ro 49, Mulguem-eup, Yangsan-si, Gyeongsangnam-do 50612, Republic of Korea; ^4^Institute of Translational Dental Sciences, Pusan National University, Busandaehak-ro 49, Mulguem-eup, Yangsan-si, Gyeongsangnam-do 50612, Republic of Korea; ^5^Department of Oral Pathology, School of Dentistry, Pusan National University, Busandaehak-ro 49, Mulguem-eup, Yangsan-si, Gyeongsangnam-do 50612, Republic of Korea

## Abstract

Bone homeostasis destruction is triggered by the uncontrolled activity of osteoblasts and osteoclasts. Targeting both the regulation of bone formation and resorption is a promising strategy for treating bone disorders. Cordycepin is a major component of Chinese caterpillar fungus* Cordyceps militaris*. It exerts a variety of biological actions in various cells and animal models. However, its function on bone metabolism remains unclear. In the present study, we discovered a dual-action function of cordycepin in murine MC3T3-E1 and RAW264.7 cells. MC3T3-E1 cells were cultured in an osteogenic medium in the presence of 1 *μ*M cordycepin for up two weeks. Cordycepin was used for effects of osteoblast and osteoclast differentiation. Cell viability was measured using the MTT assay. Osteoblast differentiation was confirmed by alizarin red staining, ALP activity, western blot, and real-time PCR. Osteoclast differentiation and autophagic activity were confirmed via TRAP staining, pit formation assay, confocal microscopy, western blot, and real-time PCR. Cordycepin promoted osteoblast differentiation, matrix mineralization, and induction of osteoblast markers via BMP2/Runx2/Osterix pathway. On the other hand, RAW264.7 cells were differentiated into osteoclast by RANKL treatment for 72 h. 1 *μ*M cordycepin significantly inhibited RANKL-induced osteoclast formation and resorption activity through disturbing the actin ring-formatted sealing zone and activating cathepsin K and MMP9. These findings indicate that cordycepin might be an innovative dual-action therapeutic agent for bone disease caused by an imbalance of osteoblasts and osteoclasts.

## 1. Introduction

Bone tissue is a highly dynamic central tissue and essential for the efficient execution of skeletal function. Human bone is continually converted to new bone over the course of one's life by a physiological process, known as bone remodeling [1, 2]. Bone homeostasis is maintained through a well-regulated balance between bone formation and bone resorption, coordinated by osteoblasts and osteoclasts, respectively [3]. An imbalance between osteoblast and osteoclast activities may trigger bone disease such as osteoporosis, osteopetrosis, Paget's disease, and rheumatoid arthritis [4].

Osteoblasts, derived from mesenchymal stem cells, are responsible for bone formation and their functions are determined by a status of differentiation. Immature osteoblasts, preosteoblastic/stromal cells, secrete RANKL (receptor activator of nuclear factor-*κ*B ligand), which requires osteoclast differentiation, whereas mature osteoblasts develop a bone matrix through collagen synthesis and mineralization via the activation of signaling proteins and transcription factors such as a BMP (bone morphogenetic proteins) signaling pathway [5–8].

Osteoclasts originate from hematopoietic stem cells are multinucleated cells differentiated by fusion of monocytes/macrophages. Osteoclast formation is triggered by the interaction of RANK (receptor activator of nuclear factor-*κ*B) and its ligand RANKL. This interaction stimulates TRAF6 (tumor necrosis factor receptor-associated factor 6), transcription factor cFOS, and NFATc1 (nuclear factor of activated T-cells cytoplasmic 1) in sequence. Mature osteoclasts induce osteoclast-specific factor upregulation, including TRAP (tartrate-resistant acid phosphatase), cathepsin K, MMP-9 (matrix metallopeptidase-9), and OSCAR (osteoclast-associated receptor) and are capable of bone resorption [9–14]. Recently, there has been much interest regarding natural ingredients or herbal medicine in the management of bone-related disease because current osteoporosis medications carry multiple concerning side effects [15, 16].

Cordycepin, 3′-[4]deoxyadenosine, is isolated from the Chinese caterpillar fungus* Cordyceps militaris* and has long been used as traditional medicine [17]. Cordycepin has been reported to have various biological properties, such as antitumor, antibacterial, and anti-inflammatory effects [18–21]. Also, cordycepin helps to protect against oxidative and inflammatory stress in osteogenesis [22, 23]. There are few studies available about the regulatory effects of cordycepin on bone metabolism mechanisms. However, the potential actions of cordycepin regarding osteoblast and osteoclast differentiation remains unclear.

This study aimed to identify cordycepin as a candidate that can control osteoblastogenesis and osteoclastogenesis. Furthermore, we report on the underlying mechanism by cordycepin that modulates BMP signaling-induced osteoblast differentiation in murine preosteoblastic MC3T3-E1 cells and the inhibition of RANKL-induced osteoclast differentiation in murine macrophage RAW264.7 cells.

## 2. Materials and Methods

### 2.1. Reagents

Fetal bovine serum (FBS), minimum essential medium alpha medium (*α*-MEM), penicillin-streptomycin, and trypsin-EDTA were purchased from GIBCO (Grand Island, NY, USA). Dulbecco's Modified Eagle medium (DMEM) was obtained from Thermo Fisher Scientific (Pittsburgh, PA, USA). Cordycepin, alizarin red S, an Alkaline Phosphatase Assay Kit, and TRAP Staining Kit were purchased from Sigma-Aldrich (St. Louis, MO, USA). RANKL was purchased from R&D systems (Minneapolis, MN, USA). 3-[4,5-dimethylthiazol-2-yl]2,5-diphenyl tetrazolium bromide (MTT) and dimethyl sulfoxide (DMSO) were obtained from DUCHEFA Biochemie (Haarlem, The Netherlands). Mouse antibodies BMP-2, Runx-2, Osterix, Osteopontin, and sRANKL were purchased from Abcam (Cambridge, UK). Antibodies of mouse TRAF6, cFOS, phospho-cFOS, NFATc1, cathepsin K, p38 MAPK, phospho-p38 MAPK, ERK1/2, phospho-ERK1/2, JNK, and phosphor-JNK were provided by Cell Signaling Technology (Beverly, MA, USA). The anti-mouse HRP-conjugated secondary antibody and anti-rabbit HRP-conjugated secondary antibody were purchased from Enzo Biochem (Farmingdale, NY, USA). TOPreal™ qPCR 2X PreMIX and M-MLV cDNA Synthesis Kit were obtained from Enzynomics (Dajeon, KOR). PCR primers (OSCAR, Ctsk1, NFATc1, GADPH) were purchased from Qiagen (Hilden, DEU).

### 2.2. Cell Culture and Osteoblast/Osteoclast Differentiation

The murine calvaria-derived MC3T3-E1 was obtained from America Type Culture Collection (Manassas, VA, USA). MC3T3-E1 cells were maintained in an alpha modification of Dulbecco's Modified Eagle's medium (*α*-MEM without ascorbic acid, GIBCO, Grand Island, NY, USA) with 10% FBS and 1% penicillin-streptomycin at 37°C in a humidified 5% carbon dioxide (CO_2_) atmosphere. For osteoblast differentiation, MC3T3-E1 cells were seeded in 24 well plates grown to 100% density. The culture medium was replaced with an osteogenic medium (MEM alpha modification containing 50 *μ*g/ml ascorbic acid, 10 mM *β*-glycerophosphate, and 0.5, 1 *μ*M cordycepin). The osteogenic medium was changed every two days for two weeks. The murine macrophage RAW264.7 was purchased from Korean Cell Line Bank (Seoul, KOR). RAW264.7 cells were cultured in a Dulbecco's Modified Eagle's medium (DMEM) with 10% FBS at 37°C in a humidified 5% carbon dioxide (CO_2_) atmosphere. For the osteoclast differentiation, cells were seeded on 24 well plates in DMEM containing 25 ng/ml RANKL for three days. The medium containing RANKL was changed every day for three days.

### 2.3. Cell Viability Measurement

The cell viability of MC3T3-E1 and RAW264.7 cells were determined using an MTT assay. Cells were cultured in 96-well plates and then incubated for different time periods in the presence of various concentrations of cordycepin (0–5 *μ*M). After terminating the treatment, the medium was removed and 100 *μ*l of MTT (500 mg/mL) was added to each well. The cells were incubated for 3 h at 37°C. The formazan crystals that formed were then solubilized in DMSO (200 *μ*l/well) by constant shaking for 10 min. The colored solution was quantified at 620 nm using an ELISA reader (Tecan, Männedorf, Switzerland). Cell viability was determined as a percent compared to the controls. All data was obtained from at least three independent experiments.

### 2.4. Alizarin Red s Staining

Upon the termination of cordycepin treatment (7 or 14 days), cells were fixed in 4% paraformaldehyde for 10 min at room temperature. Fixed cells were washed three times with distilled water, and then stained with alizarin red s solution for 10 min at room temperature. For quantification, cells were dissolved by PBS transferred to 96 well plates, and then their absorbance was measured at 550 nm using a microplate reader.

### 2.5. Alkaline Phosphatase (ALP) Activity Assay

To determine ALP activity, cells were treated with cordycepin and fixed by the same method used for alizarin red s staining. Cells were lysed with a lysis buffer (10 mM Tris-HCl (pH 7.5), 0.5 mM MgCl_2_, 0.1% Triton X-100) for 30 min and then reacted with a reaction buffer (25 mM glycine, 2 mM MgCl_2_, 5 mM pNPP) for 1 h at 37°C. The reaction was stopped by 1 M NaOH and protein absorbance was measured at 405 nm.

### 2.6. Tartrate-Resistant Acid Phosphatase (TRAP) Staining

RAW264.7 cells were seeded in 24 well plates at a density of 2×10^4^ cells/well. Cells were cultured in DMEM with RANKL and at various concentrations of cordycepin for three days. After osteoclast differentiation, cells were fixed in 4% paraformaldehyde for 10 min at room temperature, and then stained with a TRAP staining solution according to the manufacturer's instructions.

### 2.7. Immunofluorescent Staining

RAW264.7 cells were cultured in DMEM with RANKL and cordycepin on a Lab-Tek™ II Chamber Slide (Nunc; Thermo Fisher Scientific, Rochester, NY, USA). After three days, cells were fixed in 4% paraformaldehyde and then stained with rhodamine phalloidin (Invitrogen, Eugene, OR, USA) and Hoechst solution for 30 min. Fluorescent images were observed and analyzed using a Zeiss LSM 750 laser-scanning confocal microscope (Göettingen, Germany).

### 2.8. Pit Formation Assay

A pit formation assay was performed using a commercially available osteo assay plate (Corning, NY, USA). RAW264.7 cells were seeded in 24 well plates coated with calcium phosphate and incubated in DMEM with RANKL and various concentrations of cordycepin for three days. After osteoclast differentiation, mature osteoclasts were lysed by 10% NaOCl and washed with distilled water. The pit area in each plate was measured by image J.

### 2.9. Western Blot Assay

Attached cells were washed twice in ice-cold PBS, scraped, and collected by centrifugation at 8,000 rpm for 10 min. The lysates were solubilized in a 150 *μ*l RIPA buffer (300 mM NaCl, 50 mM Tris-Cl [pH 7.6], 0.5% Triton X-100, 2 mM PMSF, 2 *μ*l/ml aprotinin and 2 *μ*l/ml leupeptin, protease inhibitor cocktail) incubated at 4°C for 1 h and centrifuged at 13,200 rpm for 30 min at 4°C. Protein concentrations of cell lysates were determined using a Bradford protein assay (Bio-Rad, Richmond, CA, USA), and 20 *μ*g of proteins were resolved by 10% SDS/PAGE gel. The gel was transferred to polyvinylidene fluoride (PVDF) membranes (Millipore, Billerica, MA, USA). After 5% nonfat milk blocking, the membranes were reacted with appropriate primary antibodies. Immunostaining with secondary antibodies was detected using the Chemiluminescent HRP Substrate Kit (Millipore, Billerica, MA, USA) and SuperSignal West Femto substrate (Pierce, Rockford, IL, USA).

### 2.10. Real-Time RT-PCR

Gene expression was analyzed with real-time reverse transcription-polymerase chain reaction (RT-PCR). Total RNA was isolated from RAW264.7 cells using a RNeasy Mini Kit (Qiagen, Hilden, DEU). Total RNA concentration was determined with a NanoDrop 2.0 spectrophotometer (Thermo Fisher Scientific, Pittsburgh, PA, USA), and 2 *μ*g total RNA was reversibly transcribed into cDNA for 5 min at 70°C using a M-MLV cDNA Synthesis Kit (Enzynomics, Dajeon, KOR). PCR was performed using the TOPreal™ qPCR 2X PreMIX (Enzynomics, Dajeon, KOR) and run on an ABI 7500 Fast Real-Time PCR System (Applied Biosystems 7500 System, Foster City, CA, USA; using Sequence Detection System software version 2.0.1). The relative mRNA levels were normalized using GAPDH as a housekeeping gene.

### 2.11. Statistical Analysis

Reported data represents mean ± SD (standard deviation). Statistical analyses were conducted via one-way ANOVA followed by Dunnett's multiple comparison test. Differences with* p *values < 0.05 were considered significant.

## 3. Results

### 3.1. Cordycepin Has No Cytotoxicity in Murine Preosteoblastic MC3T3-E1

**Figure 1:**
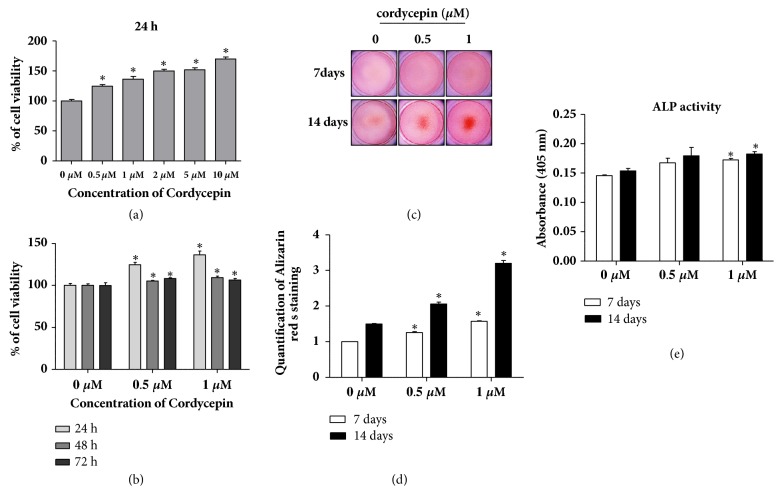
The effect of cordycepin on MC3T3-E1 cell viability and osteogenic differentiation. (a) Cells were treated with cordycepin (0-5 *μ*M) for 24 h. (b) Cells were incubated for 24 h, 48 h, and 72 h in the presence of 0.5 and 1 *μ*M cordycepin. (c) Mineralized osteoblast nodules formation was detected using alizarin red s staining. Cells were cultured in an osteogenic medium containing 0.5 and 1 *μ*M cordycepin for seven and fourteen days. (d) Quantification of alizarin red s staining was performed after extraction with ethylpyridinium chloride. (e) ALP activity was performed after cordycepin treatment under the same conditions as described in (c). Absorbance was measured at 405 nm. The medium was changed every three days. Error bars (a-e) denote mean ± SD (standard deviation). *∗P *< 0.05 versus control, respectively.

To investigate the cytotoxic effect of cordycepin, murine preosteoblastic MC3T3-E1 cells were cultured with 0-5 *μ*M cordycepin for 24 h. After treatment, cell viability was determined using an MTT assay. As shown in Figure 1(a), cordycepin alone was noncytotoxic to cells at the indicated concentrations. Rather, the viability of MC3T3-E1 increased in a dose-dependent manner. Cells were also treated with 0.5 and 1 *μ*M cordycepin-low concentrations, for 24, 48, and 72 h with no cytotoxic effects observed in MC3T3-E1 (Figure 1(b)).

### 3.2. Acceleration of Osteoblast Differentiation/Mineralization in MC3T3-E1 by Cordycepin

**Figure 2:**
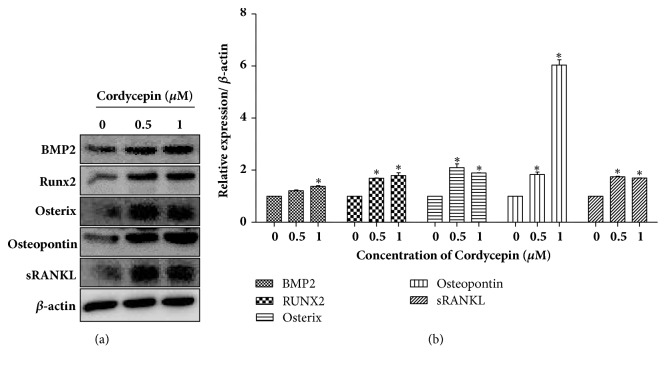
Protein expression levels of osteoblast marker (BMP2, Runx2, Osterix, Osteopontin, and sRANKL) after cordycepin treatment in MC3T3-E1. (a) Cells were incubated in an osteogenic medium with 0.5 and 1 *μ*M cordycepin for 24 h. (b) Relative protein expression levels were normalized to *β*-actin. Error bars represent mean ± SD. *∗P *< 0.05 versus control, respectively.

To determine whether cordycepin affected osteoblast differentiation and mineralization, alizarin red S staining and an ALP activity assay were performed. Cells were treated with 0.5 and 1 *μ*M of cordycepin in an osteogenic medium for seven and fourteen days. As shown in Figure 1(c), the control group had no mineralized nodules, whereas the treated group indicated substantial staining of alizarin red s in a dose-dependent manner. The quantitative strength of alizarin red s staining was in accordance with its image patterns (Figure 1(d)). The staining intensity was especially strong after fourteen days compared to after seven. In the same conditions as described in alizarin red S staining, ALP activity increased in both dose- and time- dependent manners (Figure 1(e)). Next, we discovered the expression of osteoblast biomarker proteins, as shown in Figure 2. Cordycepin significantly promoted upregulation of BMP2 protein expression. Cordycepin also increased the expression of BMP downstream cascade, Runx2, and Osteopontin. Another osteoblast differentiation transcription factor, Osterix, was upregulated dose dependently. Cordycepin was found to enhance RANKL secretion for essential osteoclast differentiation factor in MC3T3-E1 cells. Collectively, these results signified that cordycepin effectively accelerated the differentiation of preosteoblastic MC3T3-E1 cells into active osteoblasts.

### 3.3. Cordycepin Has No Cytotoxicity in Murine Macrophage RAW264.7

**Figure 3:**
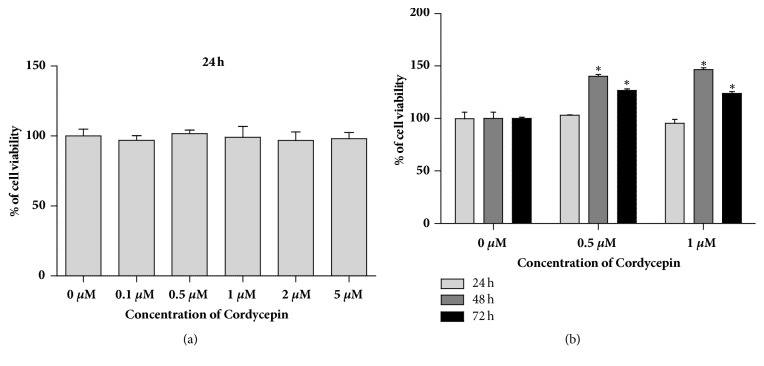
The effect of cordycepin in RAW264.7 cell viability. (a) Cells were treated with cordycepin (0–5 *μ*M) for 24 h. (b) Cells were incubated for 24 h, 48 h, and 72 h in the presence of 0.5 and 1 *μ*M cordycepin. Data represents the means ± SEM of three independent experiments. *∗P *< 0.05 versus control, respectively.

To confirm the cytotoxic effect of cordycepin, murine macrophage RAW264.7 cells were cultured with 0-5 *μ*M cordycepin for 24 h. After treatment, cell viability was determined using an MTT assay (Figure 3). As shown in Figure 3(a), cell viability was not affected by cordycepin at the indicated concentrations. Also, cells were treated with 0.5 and 1 *μ*M cordycepin-low concentrations, for 24, 48, and 72 h with no noted cytotoxic effects on RAW264.7 (Figure 3(b)).

### 3.4. Attenuation RANKL-Induced Osteoclastogenesis in RAW264.7 by Cordycepin

**Figure 4:**
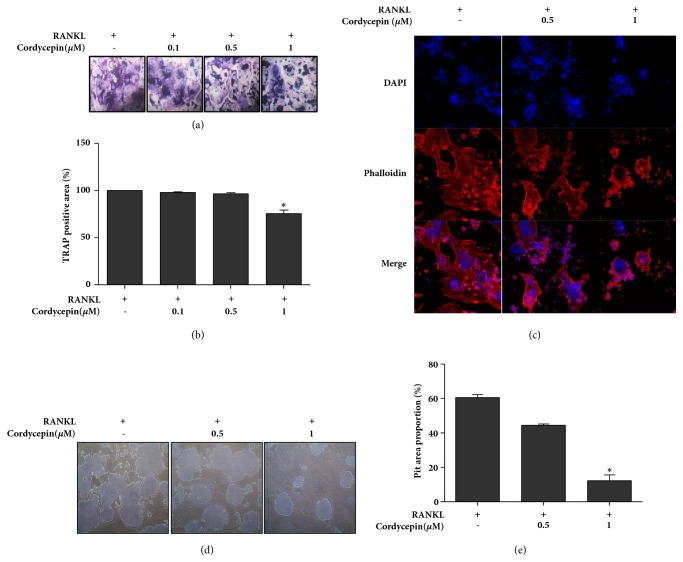
Inhibitory effect of cordycepin on RANKL-induced osteoclast differentiation in RAW264.7 cells. Cells were exposed to 25 ng/ml RANKL for 72 h in the absence or presence of cordycepin. (a) Multinucleated osteoclasts were stained by using a commercially available TRAP assay kit. Osteoclasts were observed under light microscopy (magnification: 100-fold). (b) TRAP-positive multinucleated osteoclasts occupying the area were measured by image J. Cells were counterstained with hematoxylin solution. (c) Differentiated osteoclasts were fixed and stained with DAPI (nuclei) and rhodamine phalloidin (F-actin). Original magnification: 100-fold. (d) Cells were treated with 25 ng/ml RANKL in the absence or presence of the indicated concentration of cordycepin for 72 h on calcium phosphate-coated plates. Pits were observed under light microscopy (magnification: 100-fold). (e) The graph represents the resorption pit area. Data represents the means ± SEM of three independent experiments. *∗P *< 0.05 versus positive control.

In the next phase, we formed a hypothesis that cordycepin can affect RANKL-induced osteoclast differentiation. TRAP is a marker that directly indicates osteoclast activity. RAW264.7 cells were exposed to 25 ng/ml RANKL both with or without different cordycepin concentrations. Groups were set to concentrations of 0 *μ*M, 0.1 *μ*M, 0.5 *μ*M, and 1 *μ*M for 72 h. Afterwards, TRAP staining was executed to evaluate the effects of cordycepin on RANKL-induced osteoclast differentiation. As shown in Figures 4(a) and 4(b), RANKL-induced TRAP-positive osteoclast formation and cordycepin dose dependently decreased the number and area of osteoclasts.

Bone resorption is triggered when osteoclasts attach to the bone matrix and form a sealing zone. F-actin ring structure is observed at the ruffled border of mature osteoclasts and this structure can adhere to the bone's surface and then form a sealing zone [24–27]. Immunofluorescent staining was performed to examine whether cordycepin negatively regulates F-actin ring formation in osteoclasts. As expected, RANKL-exposed RAW264.7 cells formed multinucleated osteoclasts with dense and wide phalloidin-positive F-actin rings. Cordycepin treated groups, less multinucleated osteoclasts, and F-actin rings were detected in a dose-dependent manner compared to RANKL only treated group (Figure 4(c)). Next, a pit assay was conducted to ascertain the suppression effect of cordycepin on mature osteoclast resorptive activity. RAW264.7 cells were seeded on calcium phosphate-coated plates and then treated with RANKL in the absence or presence of cordycepin for 72 h. Analysis of the pit assay showed that the osteoclast-resorbed pit area's proportion of area was significantly attenuated in the presence of cordycepin, especially at the concentration of 1 *μ*M compared to the group comprising only of RANKL treated cells (Figures 4(d) and 4(e)). The data suggests that the application of cordycepin effectively suppressed RANKL-induced osteoclast differentiation, maturation, and resorption function.

### 3.5. Effect of Cordycepin on Osteoclast Marker Expression in RANKL-Stimulated RAW264.7

**Figure 5:**
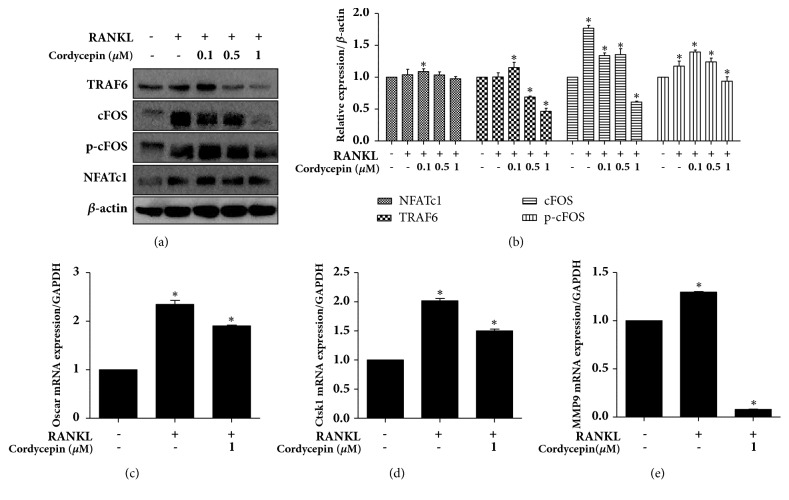
Cordycepin inhibits RANKL-mediated induction of marker expression in osteoclastogenesis. RAW264.7 cells treated with 25 ng/ml RANKL with or without cordycepin (0.1, 0.5, and 1 *μ*M) for 72 h. (a) Protein expressions of TRAF6, cFOS, phospho-cFOS, and NFATc1 were analyzed by western blot analysis. (b) Relative protein expression levels compared to *β*-actin were analyzed by image J. OSCAR (c), cathepsin K (d), and MMP9 (e) relative mRNA expression levels were analyzed by real-time RT-PCR.

**Figure 6:**
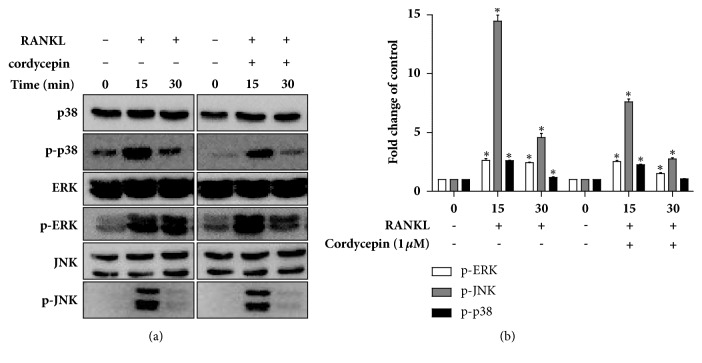
Cordycepin inhibits RANKL-mediated phosphorylation of MAPK signaling (p38, ERK, and JNK). RAW264.7 cells were stimulated with RANKL (25 ng/ml) in the absence or presence of 1 *μ*M of cordycepin for 0–30 min.

To further clarify the molecular mechanism of cordycepin's inhibition effect on osteoclast differentiation, we examined the expression levels of the osteoclast markers (TRAF6, cFOS, phospho-cFOS, NFATc1, OSCAR, cathepsin K, and MMP9) using a western blot analysis and real-time RT-PCR. The NFATc1 is a well-known master transcription factor of osteoclast development. NFATc1 and its upstream factor, TRAF6, cFOS, and phosphor-cFOS expressions were upregulated upon exposure to 25 ng/ml RANKL; 1 *μ*M cordycepin exerted a prominent inhibitory effect on these protein levels (Figures 5(a) and 5(b)). Cathepsin K and MMP9, which are major bone-digestion enzymes, are responsible for bone matrix resorption. We further examined the effect of cordycepin on OSCAR, cathepsin K and MMP9 mRNA expression levels, where 1 *μ*M cordycepin significantly downregulated RANKL-induced OSCAR, cathepsin K and MMP activation (Figures 5(c), 5(d) and 5(e)). Plus, NFATc1 and cFOS activation were caused by MAPK (mitogen-activated protein kinases) signaling, which is required for early osteoclast differentiation [28, 29]. To confirm the molecular mechanism of cordycepin effects on MAPKs following the activation of RANKL in RAW264.7 cells, we investigated the phosphorylation of p38, ERK and JNK using a western blot. Cells were treated with RANKL both with or without 1 *μ*M cordycepin for 0-30 minutes. As shown in Figure 6, RANKL-stimulated phosphorylation of p38, ERK and JNK was decreased by treatment with cordycepin.

## 4. Discussion

Myriad drugs have been used to treat bone diseases like osteoporosis, osteopetrosis, and Paget's disease. Despite their positive effectiveness on bone disorders, most of these drugs carry concerning side effects [24]. For example, bisphosphonates have been widely used to manage bone disease, but they are reported to induce severe side effects such as BRONJ (bisphosphonate-related osteonecrosis of the jaw) and esophageal irritation [25–27]. Estrogen and raloxifene therapy for osteoporosis is associated with thromboembolism [4]. Considering these apprehensions, it is necessary to search for more effective alternative agents to regulate bone metabolism without side effects. The ideal drug for bone disease treatment would improve bone quality by promoting the differentiation of osteoblasts and inhibiting osteoclastogenesis and osteoclast activity.

Several research studies have reported that cordycepin plays a function in a multitude of pathologic and physiologic processes, including tumor metastasis, vascular disorder, and brain function [28–31]. Regarding bone metabolism, cordycepin has been used to prevent oxidative stress-induced bone loss [23, 32]. However, cordycepin has rarely been studied regarding osteoblast and osteoclast differentiation. In this study, we substantiated that the natural source, cordycepin, synchronously stimulated osteoblast differentiation, mineralization, and suppressed osteoclast formation and resorption activity in vitro.

The BMP family is widely known as a regulator of morphogenesis and differentiation in various tissue [33], with BMP2 having a particularly strong osteogenic ability [34, 35]. BMP signaling stimulates osteoblast differentiation and bone formation activity via upregulation of Runx2 and Osterix, which are required transcription factors for osteogenesis and Osteopontin, a bone matrix protein that induces osteoblastic mineralization [36, 37]. Several studies reported that natural substances and herbal medicines enhance bone formation by BMP signaling stimulated osteoblast differentiation [38–40]. Thus, we hypothesized that cordycepin, which is a natural Chinese substance, may regulate osteoblast differentiation through BMP signaling. As a result, cordycepin played an important role in bone formation from preosteoblastic MC3T3-E1 cells to osteoblasts and subsequently produced bone nodule mineralization by increasing the protein expression levels of BMP2, Runx2, Osterix, and Osteopontin. RANKL secretion essential for osteoclast formation was also promoted by cordycepin treatment in MC3T3-E1 cells.

Preosteoblastic cells produce RANKL that bind to its specific receptor RANK on the surfaces of macrophages. This binding causes the activation of downstream molecules, such as TRAF6, MAPKs, and subsequently cFOS and NFATc1, which are master transcription factors for osteoclastogenesis [5, 41]. In the present study, we demonstrated that cordycepin inhibited osteoclastogenesis as induced by RANKL through TRAP staining and the measurement of osteoclast marker protein expressions. TRAP, which is an enzyme secreted by osteoclasts, has plenty of biological activity like collagen synthesis/destruction and the degradation of skeletal phosphoproteins [42, 43].

Mature osteoclasts have resorption activity characterized by a ruffle border, attachment, and a secretion phase. Differentiated osteoclasts attach to the bone's surface, and the ruffle border secretes proteolytic enzymes including cathepsins and MMP (matrix metalloproteases), which degrade and solubilize the bone mineral and collagen matrices. These resorption processes trigger within an F-actin ring organized sealing zone [44, 45]. In this study, to identify the resorptive function of osteoclasts, F-actin-specific rhodamine phalloidin staining, pit assay, and detection of cathepsin K expressions were investigated via confocal microscopy, osteo assay plates, western blot analysis, and real-time RT-PCR. The intensity of the rhodamine phalloidin-positive actin ring structure and pit area proportion were attenuated by cordycepin treatment. RANKL-increased protease cathepsin K protein and mRNA expressions were also reduced by cordycepin.

Many kinases are stimulated by the RANKL-RANK interaction, including MAPK, I*κ*B kinase, and TAK1 (MAPK kinase kinase) [46]. Among these, MAPK signaling is an important element for osteoclast differentiation and cathepsin K expression [47]. MAPK family members are composed of p38-MAPKs, extracellular signal-regulated kinases (ERK), and c-Jun N-terminal kinases (JNK) [48, 49]. We found that cordycepin suppressed RANKL-induced MAPK family member phosphorylation during early osteoclastogenesis in a short response time. Hence, these molecules might be inappropriate targets for long-term therapies targeting the treatment of bone disorders. However, the discoveries have been reported recently that regulation of preosteoclast is more effective strategy about bone disease. Preosteoclasts hardly resorb bone matrix and they are simultaneously beneficial for angiogenesis that affected osteoporosis. For this reason, preosteoclast now got more attention [50, 51]. If additional preosteoclast-related experiment is performed, it will provide a basis for the development of new bone disease treatment strategy.

## 5. Conclusions

The present study demonstrated that cordycepin accelerated osteoblast differentiation and matrix mineralization by the induction of BMP2-Runx2-Osterix signaling in MC3T3-E1 cells. In addition, cordycepin attenuated osteoclastogenesis through inhibition of the RANKL/TRAF6/MAPK pathway, resulting in the abrogation of cFOS/NFATc1/cathepsin K osteoclast marker gene expression. However, further in vivo studies are necessary to establish an accurate role and safety of cordycepin in bone metabolism. If cordycepin's capability to regulate bone remodeling is proven in animal bone models, cordycepin could be a potential agent to utilize in clinical studies for the treatment of bone loss.

## Data Availability

The data used to support the findings of this study are included within the article.
